# Stearic acid-rich interesterified fat and trans-rich fat raise the LDL/HDL ratio and plasma glucose relative to palm olein in humans

**DOI:** 10.1186/1743-7075-4-3

**Published:** 2007-01-15

**Authors:** Kalyana Sundram, Tilakavati Karupaiah, KC Hayes

**Affiliations:** 1 Food Technology & Nutrition Research Unit, Malaysian Palm Oil Board, Kuala Lumpur, Malaysia; 2 Faculty of Allied Health Sciences, National University of Malaysia, Malaysia; 3 Foster Biomedical Research Lab, Brandeis University, Waltham, MA, USA

## Abstract

**Background:**

Dietary *trans*-rich and interesterified fats were compared to an unmodified saturated fat for their relative impact on blood lipids and plasma glucose. Each fat had melting characteristics, plasticity and solids fat content suitable for use as hardstock in margarine and other solid fat formulations.

**Methods:**

Thirty human volunteers were fed complete, whole food diets during 4 wk periods, where total fat (~31% daily energy, >70% from the test fats) and fatty acid composition were tightly controlled. A crossover design was used with 3 randomly-assigned diet rotations and repeated-measures analysis. One test fat rotation was based on palm olein (POL) and provided 12.0 percent of energy (%en) as palmitic acid (16:0); a second contained trans-rich partially hydrogenated soybean oil (PHSO) and provided 3.2 %en as trans fatty acids plus 6.5 %en as 16:0, while the third used an interesterified fat (IE) and provided 12.5 %en as stearic acid (18:0). After 4 wk the plasma lipoproteins, fatty acid profile, as well as fasting glucose and insulin were assessed. In addition, after 2 wk into each period an 8 h postprandial challenge was initiated in a subset of 19 subjects who consumed a meal containing 53 g of test fat.

**Results:**

After 4 wk, both PHSO and IE fats significantly elevated both the LDL/HDL ratio and fasting blood glucose, the latter almost 20% in the IE group relative to POL. Fasting 4 wk insulin was 10% lower after PHSO (p > 0.05) and 22% lower after IE (p < 0.001) compared to POL. For the postprandial study the glucose incremental area under the curve (IAUC) following the IE meal was 40% greater than after either other meal (p < 0.001), and was linked to relatively depressed insulin and C-peptide (p < 0.05).

**Conclusion:**

Both PHSO and IE fats altered the metabolism of lipoproteins and glucose relative to an unmodified saturated fat when fed to humans under identical circumstances.

## Background

Partially hydrogenated oils represent a means of removing unstable polyunsaturated fatty acids (PUFA) from fats in foods that require longer shelf life. Although semisolid at room temperature, such products also technically represent a monounsaturated fatty acid (MUFA) replacement for natural saturated fatty acids (SFA). Unfortunately, partially hydrogenated oil represents the prime source of *trans *fatty acids (TFA) in the human diet. The negative effects of TFA on lipoproteins have been widely publicized [1], which led to inclusion of TFA on food labels by various governments. Interesterification (fatty acid randomization) of fat is fast becoming an alternate modification technique, whereby insertion of saturated fatty acids (SFA), typically as stearic acid (18:0), is employed to harden an oil to a plasticity comparable to earlier *trans *fat preparations or to hardness of a natural saturated fat. The assumption has been that since 18:0, as consumed in natural fats at 2–4% of daily energy, is thought to have a neutral effect on metabolism of cholesterol and lipoproteins [2-4], the response to 18:0 at any level of intake and in any triacylglycerol (TG) configuration would be neutral. However, like partial hydrogenation, the random insertion of fatty acids on the glycerol backbone of the fat molecule associated with chemical interesterification alters natural TG structure, referred to as TG molecular species or TG-MS [5]. Several studies reveal that altering dietary TG-MS, ie. the natural position of specific fatty acids on the 3C glycerol backbone (carbons designated as sn1, sn2, and sn3), affects both lipoprotein metabolism and experimental atherogenesis [6-9]. On the other hand, in certain situations serum lipid parameters were minimally affected when the intake of randomized fat was modest or acute, as in a single meal challenge with a modified fat containing altered TG-MS [10-12].

Accordingly, we hypothesized that chemically modified vegetable oils, either partially hydrogenated or interesterified with abundant 18:0, might not have the same metabolic impact as a naturally-configured saturated oil rich in palmitic (16:0) + oleic (*cis*18:1) fatty acids. Three fats were prepared as suitable hardstock for margarine and other solid fat formulations. The natural fat was based on palm olein (POL), used extensively for many food formulations that still require a degree of fat saturation. Partially hydrogenated soybean oil (PHSO) was included as the typical example of a commonly used semisolid fat containing geometric fatty acid isomers (*cis *or *trans*) of 18:1 and 18:2 (linoleic acid). The third fat was chemically interesterified (IE), which randomly incorporated 18:0 into regular TG molecules in soybean oil as an alternative hard fat for food formulations. The primary focus was on blood lipids and the lipoprotein profile in normolipemic humans, with a secondary focus on blood glucose, insulin and C-peptide.

## Methods

### Study design

The study, approved by the Program Advisory Committee at the Malaysian Palm Oil Board, Bangi, Malaysia, included informed signed consent by the human volunteers. The 3 dietary fats were compared using a crossover design with 4 wk for each fat. Specifically, diets were identified as A, B, or C, and subjects were assigned to each in random order until all three had been consumed by each person. Thus, all three diets were available during each 4 wk treatment period, but individual assignment to each was randomized to help negate possible carryover effects. Subjects were advised to maintain their usual food intake for all test rotations, which were identical in all aspects except for the cooking fat. An electronic weighing balance in the food service area was used to weigh food portions. Blood samples were collected prior to beginning a test period and twice during the last week (averaged as the terminal value).

Within each 4 wk period, an identical subgroup of 19 subjects participated in an acute *postprandial challenge *with their respective test fat after 2 wk into each diet rotation. Thus, volunteers were preconditioned with the test fat during the 14 d prior to the postprandial challenge. After an overnight fast of at least 12 h, volunteers reported to the laboratory on the 15th morning. Body weight was recorded and baseline fasted blood was drawn. Volunteers then consumed a standardized test meal comprising weighed portions of fried rice, fried potatoes, a slice of papaya and tea. This test breakfast contributed a total of 53 g test fat, and consumption of this meal was completed within 20 minutes of the first (baseline, 0 h) blood sampling. Blood samples were then taken sequentially at 2, 4, 5, 6 and 8 h after the test meal was consumed. Volunteers abstained from consuming any food during this 8 h period, except bottled mineral water. They also refrained from any strenuous activity during this interval. Following the 8 h sample, volunteers received a fully cooked meal.

### Study subjects

Eligibility criteria included absence of family history for atherosclerosis or hypertension, as well as no use of tobacco or alcohol or adherence to any weight loss program or prescribed medication. A total of 11 healthy men and 21 women (ave age 30 ± 8 y) were initially recruited by advertisement among the 450 staff members at the research facilities of the Palm Oil Board. One subject was unable to comply with the dietary protocol and subsequently was dropped from the study, while another did not complete the third rotation due to acute appendicitis. The baseline demographics of the remaining 30 fasting subjects who were recruited after initial screening and who successfully completed the study were as follows (mean ± SD): BMI 22 ± 4; body wt 56 ± 10 kg; blood lipids (total cholesterol, 5.05 ± 0.53 mmol/L; LDL-C, 3.17 ± 0.51 mmol/L; HDL-C mmol/L, 1.48 ± 0.25 mmol/L; TG, 0.89 ± 0.30 mmol/L) and plasma glucose concentrations (5.43 ± 0.29 mmol/L).

### Test fats

The 3 test fats (Table 1) included palm olein, as a natural dietary fat (POL); a partially hydrogenated soybean oil (PHSO); and an interesterified fat (IE) prepared in a 2-step procedure. Palm olein is the liquid to semisolid oil fraction obtained when palm oil is melted, followed by rapid cooling to separate the upper liquid palm olein layer (approximately 70%) from the lower solid stearin fraction (approximately 30%) by a physical fat modification process termed fractionation. POL contains more monounsaturated *cis*18:1 and less16:0 and 18:0 saturates relative to original palm oil, which has more saturated 16:0 than monounsaturated *cis*18:1. Both of the chemically modified fats, namely PHSO and IE, typically have less PUFA and *cis*18:1 content compared to native soybean oil (SBO), but have higher solid fat content to confer more plasticity and less susceptibility to rancidity. These three fats each provided a TG structure and plasticity that renders them suitable for use in common solid fat-containing food formulations.

**Table 1:** Fatty acid profiles for individual test fats and overall FA profile of test diets

**Fatty acid**	**Test oils (% total fat)**			**Test diets (% energy)**		
	
	**POL**^**1**^	**PHSO**^**2**^	**IE**^**3**^	**POL**^**1**^	**PHSO**^**2**^	**IE**^**3**^
Saturates	44.05	29.32	58.61	13.66	9.09	18.17
12:0	0.23	0.22	nd	0.07	0.12	nd
14:0	0.90	0.40	0.29	0.28	0.17	0.09
16:0	38.82	21.18	17.66	12.03	6.53	5.48
18:0	4.10	7.19	40.25	1.27	2.18	12.48
20:0	nd	0.33	0.41	nd	0.09	0.13

Monounsaturates	43.77	41.06	18.90	13.57	12.41	5.86
18:1 (n-9)	43.77	41.06	18.90	13.57	12.41	5.86

Polyunsaturates	11.58	18.79	22.48	3.59	5.79	6.97
18:2 (n-6)	11.23	17.57	20.98	3.48	5.36	6.50
18:3 (n-3)	0.35	1.22	1.50	0.11	0.43	0.47

Total *Trans*	nd	10.28	nd	nd	3.19	nd
*t*18:1n9	nd	2.26	nd	nd	0.70	nd
*t*18:1n11	nd	2.35	nd	nd	0.73	nd
*t*18:1n13	nd	1.0	nd	nd	0.31	nd
Unid	nd	4.67	nd	nd	1.45	nd

*P/S ratio	0.26	0.64	0.38	0.26	0.64	0.38

^1^POL, palm olein; ^2^PHSO, partially hydrogenated soybean oil; ^3^IE, interesterified fat (see text for details of fat composition)

To prepare the IE fat, a first-step full hydrogenation of refined SBO converted all unsaturated C18 fatty acids to 18:0, which was then blended with refined SBO and POL that yielded the target fatty acid composition. The blend was then subjected to chemical interesterification. To prepare the PHSO, refined SBO was first partially hydrogenated using standard catalytic hydrogenation to obtain a targeted amount of TFA in the oil. For the final PHSO composition, 40% of this partially hydrogenated SBO was blended with 30% refined SBO and 30% POL.

The final test fats had the following slip melting points (POL, 24.0; PHSO, 38.5; IE, 43.0°C). At 20°C the % solids equaled 20, 20 and 43 for POL, PHSO and IE, respectively. The final test diet supplying POL provided 12.0 %en as 16:0, while the PHSO fat yielded a TFA content of about 10% by wt, but contributing 3.2 %en as TFA plus 6.5 %en as 16:0 when incorporated into the final diet. The zero-*trans*, IE-fat diet provided 12.5 %en as 18:0 (Table 1).

### Test diets

Diets were prepared by a caterer who received detailed instruction from the research dietitian about the menu plan, portion size, and procedures for incorporating the test fats. A uniform menu was utilized for all 3-diet periods, which differed only in the type of test fat incorporated. Three meals a day, comprising breakfast, lunch and high tea were provided for the 4-wk period of each fat rotation. The menu, which was used to prepare the meals from Monday through Saturday noon, was constructed according to a fixed meal plan: For breakfast, a rice or noodle dish and a snack item cooked with the test fat was served with either coffee or tea. Lunch included either fish or chicken and 2 vegetables cooked with the test fat and accompanied by rice and fruits along with either tea or coffee. For high tea, a fried snack item incorporated the test fat, which was served with either tea or coffee. Because the subjects consumed their off-campus evening meal and Sunday meals with their families at home, they were provided with the appropriate cooking fat to incorporate into home meal preparations during each dietary period.

Fat and protein content of weighed food portions were determined by established AOAC methods (22). Energy value of the diets was determined with an automated bomb calorimeter [C5000 IKA-Calorimeter system, IKA^® ^WERKE, KG, Germany]. Diets contained about 31 %en from fat, 3.5–7 %en as linoleic acid, with each test fat providing more than 70% of the total fat under controlled conditions. Dietary carbohydrate provided approximately 54 %en, and protein approximately15 %en. Carbohydrate content was indirectly estimated by difference of total protein and total lipid mass from the dried mass of the food samples. In addition, the use of the cooking fat in homes (including Sundays) was recorded in a diary to serve as an additional compliance marker. Overall, subjects were blind to the test fat fed during each test rotation.

### Blood samples

Twenty-ml blood samples were collected at baseline and on days 28 and 29 of each test-fat rotation after overnight fasting. For the postprandial study 12-ml blood samples were collected (fasting baseline, and 2,4,5,6 and 8 h). Vaccutainer^® ^tubes [Becton Dickinson Vaccutainer, NJ, USA] with or without EDTA (0.117 ml of 15% EDTA) were used for plasma and serum preparations, respectively. For serum preparation, blood was allowed to clot at room temperature for exactly 2 h. The Vaccutainer tubes were then centrifuged at 3000 × g for 20 min at 4°C to separate the serum or plasma, which was aliquoted for various lipid and lipoprotein analyses, snap-frozen with liquid nitrogen and stored at -80°C for subsequent analyses. To reduce intra-assay variation, samples from all 3 arms of dietary treatment were analyzed in a single batch.

### Analyses of lipids and lipoproteins

Cholesterol and TG concentrations in plasma were analyzed by the enzymatic procedures of Allain et al [13] and Nägele et al. [14], respectively. HDL-C determination was based on a 2-step methodology, which required first precipitating LDL and VLDL from plasma with dextran and magnesium sulfate and then assaying the supernatant containing the HDL for cholesterol [15,16]. All assays were performed using a Ciba-Corning 550 Express Autoanalyzer [Ciba-Corning Diagnostics Corp., Oberlin, Ohio, USA]. All reagents, calibrators and controls were supplied by Bayer Corp. (Tarrytown, NY, USA), HDL-C precipitant by Chiron Diagnostics Corp. [E. Walpole, MA, USA/Bayer Corp., Tarrytown, NY, USA]. LDL-C and VLDL-C were calculated using Friedewald's equations [18].

TG-MS composition was determined by reverse-phase HPLC. The fat blends were used directly and not subjected to further purification prior to the analysis. The mobile phase was acetone/acetonitrile (Merck) at a gradient composition beginning with 65% acetone and increasing to 85% acetone in 30 min. The mobile phase flow rate was 1.5 mL/min. Two commercially packed Genesis C18 HPLC columns (15 cm length × 4.6 mm i.d.) of 4 um particle size (Jones Chromatography, Mid Glamorgan, United Kingdom) were used to separate the TG-MS. The TG-MS were detected by an Evaporative Light Scatter Detector, ELSD. Individual peaks were identified by comparing the retention times with those of pure TG-MS standards and common vegetable oils of known TG-MS composition [17].

### Determination of plasma glucose, serum insulin and C-peptide

Plasma glucose was assessed from frozen samples by the glucose oxidase method, a 2-step enzymatic procedure [19]. A reagent kit for the enzymatic procedure, SERA-PAK^® ^Glucose (which was fully Bayer Corporation, Tarrytown, NY, USA) was used in the analysis, which was automated using the Ciba-Corning 550 Express Autoanalyzer (Ciba-Corning Diagnostics Corporation, Ohio, USA). Serum insulin was measured [20] for 0 wk, 4 wk, and postprandial samples by automated immunoassay with an IMMULITE 1000 Analyzer (Euro/DPC Limited Diagnostics Products Corporation, Los Angeles, Ca.) and expressed as uU/ml. Circulating C-peptide was measured in postprandial serum as an index of insulin secretion [21]. Samples were diluted (1:4) prior to immunoassay with the IMMULITE 1000 Analyzer. All components (chemiluminescent substrate, controls, standards and diluent) for the insulin and C-peptide immunoassays were supplied in IMMULITE kits. Results are expressed as pmol/L.

### Compliance measurements

General compliance to diet was assessed by body weight changes and the comparison between the fatty acid composition of diets eaten and plasma TG. In the first instance, weight was recorded for every subject at baseline and at weekly intervals for each test rotation. This ensured that food intake was adequate and that weight fluctuations were minimized between the test-fat rotations.

Plasma TG fatty acid composition of was determined for all subjects at the end of each test rotation. Total lipids were extracted from plasma and subjected to thin-layer chromatography to separate TG and cholesteryl esters [22,23]. In addition, fatty acids from diets as eaten were assessed following Soxhlet extraction and conversion of extracted lipids into fatty acid methyl esters for analysis by gas chromatography [Perkin-Elmer Autosystem, Perkin-Elmer, Norwalk, CT]. Test fats themselves were assayed directly after conversion to methyl esters [22,23]. Results from gas chromatography of fatty acids were obtained as percentage composition (by weight), calculated by reference to standards. The fatty acid profile of meals consumed by the subjects served to check whether the research diets achieved fatty acid targets, while the plasma TG fatty acids served to check compliance with the research protocol.

### Statistical analysis

The crossover design enabled each subject to serve as his/her own control. The Statistical Package for Social Sciences, SPSS^® ^for Windows™ application (Version 11.0, SPSS Inc., Chicago, USA) was used for all required statistical analyses. The mean of values for days 28 and 29 was treated as the end of the study period. Univariate analysis was performed for linearly independent pair-wise comparisons between baseline and end values for plasma lipids, lipoproteins, glucose, and insulin, as well as for the percent change in the measured parameters for each dietary treatment. Multivariate analyses for repeated measures (ANOVA), using the general linear model, was performed for all time × diet interactions for blood glucose, insulin and C-peptide parameters following each postprandial test fat challenge. Corrected models used against baseline values were taken as true measures of change occurring during the post-absorptive period resulting from dietary treatment. Univariate analysis was performed for linearly independent pair-wise comparisons of incremental area-under-the-curve (IAUC) data for the 8-h postprandial period calculated by the trapezoidal rule [25]. Post-hoc analyses included Bonferroni's adjustment for multiple paired comparisons of estimated marginal means as well as Duncan's test for homogeneity of the effects generated by the test fat treatments. Significance was set at *P *< 0.05 for all evaluated measures.

## Results

### Weight

Initial body weights were not altered during the experiment, as weight fluctuations between test fat rotations were comparable (<0.25 kg gained per fat) and not statistically different [data not shown].

### Diet intake

Based on weighed food portions and diary records, the daily energy consumed by subjects averaged between 2100 and 2200 kcal/d. Protein intake averages were between 80–89 g/d (about 16 %en), fat between 73–77 g/d (about 31 %en), and carbohydrate between 283–292 g/d (about 53 %en) for the 3 diet periods. Food intake patterns were consistent and similar between treatments [data not shown].

### Fatty acid and TG-MS profiles

As expected, random interesterification of fully hydrogenated soybean oil with regular soybean oil led to a significant number of TG-MS in the IE test fat having 18:0 in sn2. By contrast, the POL test fat was represented by TG-MS having significant unsaturated *cis*18:1 at sn2. Specifically, the TG-MS compositions for the natural POL and IE fats (Table 2) revealed that the sn2 (number 2 carbon of glycerol) was occupied by 18:0 or 16:0 in about 15% and 6%, respectively, of the TG molecules in IE. Thus, 21% of the TG molecules in IE had a saturated fatty acid at sn2. By contrast, no TG molecules with sn2-18:0 were detected in POL; and only 9 % of all TG molecules in POL had a saturated fatty acid at sn2 (as 16:0). Thus, the total saturated fatty acid content at sn2 was more than twice as great for IE fat (sn2-18:0+16:0). By contrast, 87% of TG molecules in POL had *cis*18:1 at sn2, while only 55% TG molecules in IE fat had *cis*18:1 at sn2. Surprisingly, only 1% of TG in POL had 18:2 at sn2, compared to 21% for the IE fat; and about 20% of TG molecules in POL had 18:2 at sn1,3, whereas 42% of the TG molecules in IE had 18:2 at these end carbons. Because standards for the TFA-containing TG molecules separated by this HPLC method are not available, one can only assume that partial hydrogenation of SBO generated substantial numbers of TFA at sn2, because most of the monounsaturated fatty acids in PHSO (which would include *trans*18:1) are reportedly located at sn2 [24].

**Table 2:** Major Triglyceride Molecular Species (%) in POL and IE fats *

**TG Species**	**POL**	**IE**
LLnLn	ND	2.00
LLLn	ND	0.85
LLL	ND	0.78
PLLn	ND	0.42
OLL	0.20	2.63
PLL	0.90	4.64
OOL	0.70	2.00
SLL	ND	12.47
PPL	8.60	3.42
OOO	2.70	0.48
SOL	ND	13.79
SOS	ND	15.84
SOO	1.5	4.62
POS	4.2	15.25
PPS	ND	2.92
SSO	ND	10.81
SSS	ND	3.76
POP	39.5	ND
POO	32.2	ND
POL	9.3	ND
**TOTAL**	99.80	96.68

L = linoleic; Ln = Linolenic; O = oleic; P = palmitic; S = stearic

ND, not detected

*Triglyceride species for PHSO are not reported since they were not possible to assess due to the complexity of the TG species arising from the the lack of authentic standards to identify the various trans fatty acid isomers present.

Compliance check for test fat consumption deduced that specific FA of plasma TG (Table 3) reflected the TG fatty acid profile of both the test fat as well as the total diet fat (Table 1) for each fat rotation. Thus, plasma 16:0 and 18:2 were highest and lowest, respectively, in dietary and plasma TG during POL; 18:0 was highest in plasma TG during IE intake; and *trans*18:1 was only detected in plasma TG after feeding the PHSO diet.

**Table 3:** Fatty Acid Composition (%) of Plasma Triacylglycerol

**Fatty acid**	**Test diets**		
	
	**POL**^**1**^	**PHSO**^**2**^	**IE**^**3**^
14:0	1.4 ± 0.5^a^	1.4 ± 0.4^b^	1.1 ± 0.4^a,b^
16:0	29.5 ± 2.9^a,b^	27.2 ± 2.8^a^	26.6 ± .1.8^b^
16:1n7	3.8 ± 1.1	3.6 ± 1.4	3.5 ± 1.0
18:0	3.3 ± 0.8^a^	3.4 ± 0.5^b^	5.3 ± 2.4^a,b^
18:1	43.4 ± 4.2^a,b^	37.1 ± 3.0^a,c^	40.3 ± 3.3^b,c^
*t*18:1n9	nd	2.8 ± 0.6	nd
*t*18:1n11	nd	0.8 ± 0.3	nd
18:2	16.1 ± 4.4^a,b^	20.6 ± 5.1^a^	20.1 ± 2.8^b^
18:3	0.7 ± 0.4^a^	1.2 ± 0.5^a,b^	0.7 ± 0.2^b^
20:4n6	0.9 ± 0.3	1.0 ± 0.3	0.9 ± 0.2
22:6	1.3 ± 0.7	1.7 ± 0.7	1.3 ± 0.6

Values are means ± SD (n = twice per rotation)

^1^POL, palm olein; ^2^PHSO, partially hydrogenated soybean oil; ^3^IE, interesterified fat (see text for details of fat composition)

^a,b,c ^means with common superscripts differ, p < 0.05; nd = not detectable.

### Lipid and lipoprotein changes

Plasma analysis revealed that HDL-C and LDL-C were the only two lipid parameters significantly affected by dietary treatment, with TC and TG unperturbed (Table 4). After adjusting for multiple comparisons between dietary treatments, plasma HDL-C was significantly lower (p < 0.001) both during the PHSO (-8%) and IE (-9%) diet treatments compared to POL. Dietary fat also affected absolute change in LDL-C, with the concentration after the PHSO being 7% greater than POL (*P *< 0.05). The IE diet effect was intermediate and not significantly different from either POL or PHSO (Table 3).

**Table 4:** Fasting plasma lipid, lipoprotein cholesterol, glucose, and insulin concentrations after 4 wk of test diets

	**Test diet**		
	**POL**^**1**^	**PHSO**^**2**^	**IE**^**3**^

Total cholesterol (mmol/L)	4.93 ± 0.58	5.03 ± 0.69	4.89 ± 0.63
HDL-C (mmol/L)	1.43 ± 0.24^a,b^	1.32 ± 0.24^a^	1.30 ± 0.22^b^
% change vs POL	--	-7.6 ± 6.7	-9.1 ± 6.7
LDL-C (mmol/L)	3.08 ± 0.54^c^	3.30 ± 0.63^c^	3.20 ± 0.61
% change vs POL	--	7.2 ± 11.5	3.8 ± 9.7
VLDL-C (mmol/L)	0.42 ± 0.16	0.41 ± 0.16	0.40 ± 0.13
Triacylglycerol (mmol/L)	0.91 ± 0.34	0.88 ± 0.35	0.86 ± 0.28
TC/HDL-C	3.53 ± 0.68^a,b^	3.91 ± 0.82^a^	3.88 ± 0.83^b^
% change vs POL	--	10.8 ± 8.5	9.7 ± 9.0
LDL-C/HDL-C	2.23 ± 0.59^a,b^	2.59 ± 0.69^a^	2.56 ± 0.74^b^
% change vs POL	--	16.0 ± 12.1	14.7 ± 13.4
Plasma glucose (mmol/L)	5.62 ± 0.48^a,c^,	5.91 ± 0.58^b,c^	6.67 ± 0.70^a,b^
% change vs POL	--	5.1 ± 9.6	18.7 ± 11.3
Plasma insulin (uIU/mL)	10.11 ± 4.67^a^	9.12 ± 3.92^c^	7.93 ± 3.18^a,c^
% change vs POL	^---^	-9.7 ± 26.0	-21.6 ± 22.2

Mean ± SD ; n = 30

^1^POL, palm olein; ^2^PHSO, partially hydrogenated soybean oil; ^3^IE, interesterified fat (see text for details of fat composition)

^a,b ^means with common superscripts differ, p < 0.001. ^c ^p < 0.05

Ratios derived from absolute measurements of TC, LDL-C and HDL-C at the end of each dietary treatment (Table 3) reveal that PHSO and IE treatments both increased the TC/HDL-C ratio by about 10% relative to the POL (*P *< 0.001), but did not differ from each other. The LDL-C/HDL-C ratio was similarly increased by the two modified fats, with differences being about 15% greater than the POL diet (*P *< 0.001).

### Glucose, insulin, and C-peptide

#### 4 wk fasting data

**Figure 1:**
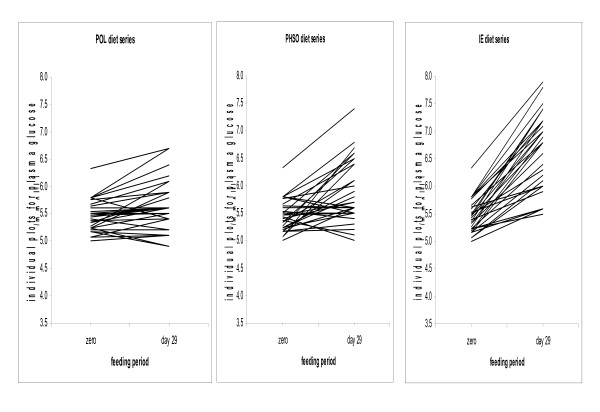
Individual fasting glucose values are depicted at entry and after 4 wk on each test fat. The rise was 3% for POL, 9% for PHSO, and 22% for IE.

Fasting plasma glucose at the end of each test fat period (Table 4), revealed a significantly higher value after IE treatment than after either POL or PHSO (p < 0.001). Glucose also increased modestly after the PHSO diet relative to POL (p < 0.05). Individual fasting glucose data measured at entry and the end of each test fat period revealed that plasma glucose for every subject increased during the IE diet period (Fig 1).

Fasting serum insulin at 4 wk varied greatly among individuals, but univariate analysis revealed significantly lower insulin values after IE (p < 0.001) with a tendency for PHSO to be lower (p < 0.10) relative to POL. Thus, the percent change for PHSO (-10%) was approximately half that observed for IE (-22%) relative to POL (Table 4). Consequently the average fasting 4 wk-insulin for each fat treatment was inversely related to the respective glucose concentration, ie. the most elevated fasting glucose following the IE period was associated with the lowest 4 wk fasting insulin, whereas the insulin value following PHSO, like glucose, was intermediate between POL and IE.

#### Postprandial responses

**Figure 2:**
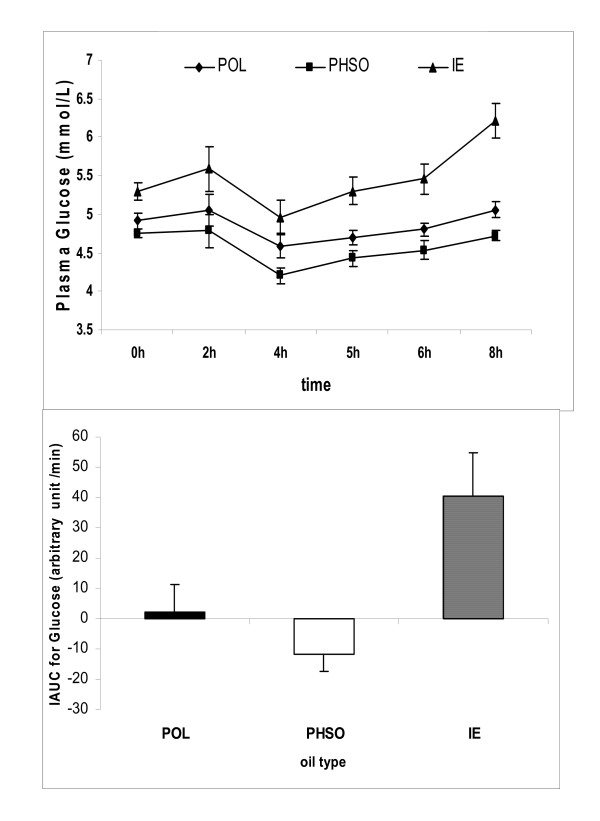
**a (top) **The relative profiles for postprandial glucose are depicted for 8 h following a challenge breakfast with either POL, PHSO, or IE fat in the test meal (means ± SE, n = 19). After 6 h and 8 h the IE glucose was significantly greater than PHSO and POL. For the entire period the IAUC for IE was 40–45% greater than the other two fats (**Figure 2b, below**) Postprandial meals were consumed at the beginning of wk3 during each diet period (means ± SE, n = 19).

In the postprandial meal challenge after 2 wk on the diet, plasma glucose became significantly elevated 6 h after the IE meal compared to POL and PHSO (p < 0.001). The latter two fats displayed similar postprandial glucose dynamics with both returning to baseline by 6 h (Fig 2a). Based in part on the slightly higher fasting glucose values for IE at the beginning of the postprandial test, the incremental area under the curve (IAUC) for glucose during the 8 h IE fat challenge was approximately 40–45% greater (p < 0.001) than the two other fats (Fig 2b).

**Figure 3:**
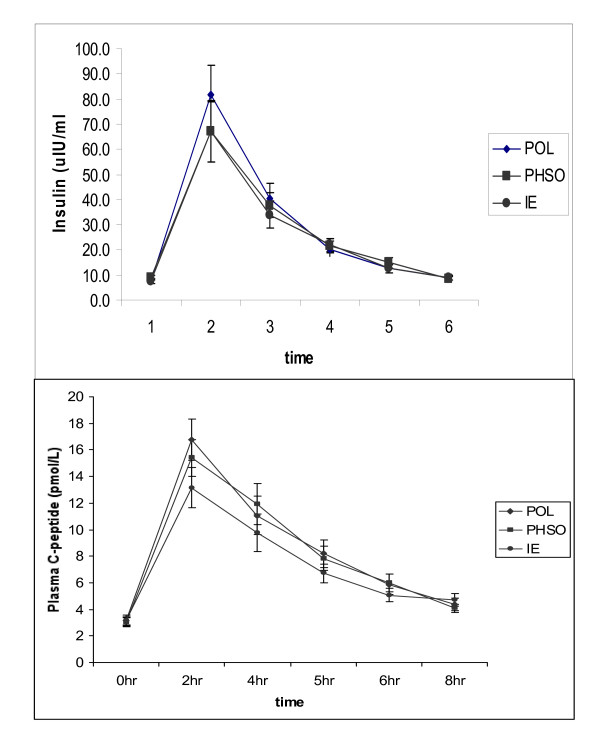
**a (top) **Postprandial plasma insulin was significantly higher for POL than either other fat after 2 h (mean ± SE). **Fig 3b (below) **Postprandial plasma C-peptide was significantly lower 2 h after the IE meal (mean ± SE).

To explore insulin dynamics postprandially, both insulin and C-peptide were measured throughout the 8 h postprandial period. Both parameters peaked sharply at 2 h, and C-peptide was directly related to the insulin concentration at this time interval (Fig 3a and 3b). The 2 h insulin was significantly lower after both the PHSO (p < 0.05) and IE (p < 0.05) challenge compared to POL, while C-peptide was significantly lowered only by IE (13.2 ± 6.6 pmol/L; p < 0.05)) compared to either POL (16.0 ± 7.4 pmol/L) or PHSO (15.6 ± 6.4 pmol/l). POL and PHSO did not differ from each other.

## Discussion

### Interesterification and lipoproteins

Based on previous data concerning TG-MS affecting lipid metabolism [5,6], a primary objective of this study was to compare the relative response by plasma lipoproteins to structural differences in dietary fatty acids and TG-MS introduced by partial hydrogenation (production of TFA) or interesterification with 18:0. In the final analysis, 15% of TG-MS in IE had 18:0 at sn2, compared to none of the TG-MS in POL having sn2-18:0. Furthermore, IE had 2.5-times more SFA at sn2 than POL; and although we were not able to make the assessment with our assay, many of the sn2-FA in PHSO are reportedly *trans*18:1 with essentially no SFA at sn2 (24). Thus, our data support previous observations that structural differences between fatty acids and TG-MS can perturb lipoprotein metabolism, eg. when TFA or 16:0 are introduced at sn2 in fat molecules where MUFA or PUFA normally reside [9-11,26]. Similarly, our data confirm that compared to a naturally structured fat, random interesterification of a polyunsaturated dietary fat with 18:0 alters its TG-MS as well as the lipoprotein metabolism encountered during its consumption [9,27].

Both partial hydrogenation and IE represent substitutes for naturally saturated dietary fats. Our study directly addressed the efficacy of those substitutions by comparing unmodified TG-MS in a naturally occurring saturated fat, POL, with PHSO and IE. Approximately equal amounts of total SFA (14–18 %en) were provided during the two saturated fat test periods, ie. with POL and IE diets, while the TFA-rich diet provided about 12 %en as SFA plus TFA. Both modified fats included specific alterations in sn2-FA. Although total plasma cholesterol was minimally affected by the 3 diets, the distribution of cholesterol among lipoproteins was altered. PHSO (representing unnatural incorporation of *trans*18:1 at sn2) elevated LDL; and both PHSO and IE (with 18:0 atypically present at sn2) lowered HDL relative to the naturally-structured POL with mainly *cis*18:1 and a lesser amount of 16:0 at sn2. The increased LDL/HDL ratio following consumption of PHSO was also significant and typical of the pattern reported during *trans*-fat consumption [8,9,28-30]. It is noteworthy that in at least two of those studies when 18:0 was interesterified into unsaturated oils and compared to *trans*18:1, *cis*18:1, or IE fat rich in 18:2 [8,9], both the added 18:0 in IE fat as well as *trans*18:1 from partial hydrogenation were found to raise LDL and lower HDL. Such results are similar to our findings, where the naturally occurring fat structure in POL served as the control fat.

The high proportion of test fat (>70%) in our diets was coupled with specific exchanges between fatty acids and modified TG-MS. The comparison was focused on 16:0 in a natural fat, 18:0 from a randomized fat, and 16:0 plus about one-third the amount of TFA from partial hydrogenation. This comparison between relatively high intakes of specific SFA and TFA enhanced the potential to detect an effect induced by the concomitant change in dietary TG-MS. Despite the fact that the TFA diet provided 50% more 18:2 than POL, and favorably increased 18:2 among plasma TG fatty acids, the TFA diet still exerted a more negative impact on plasma lipoproteins. One would predict that reducing 18:2 in the TFA diet to that of the control diet would have rendered the TFA diet even less desirable [9]; or alternatively, had the POL diet been adjusted to contain an equivalent amount of 18:2 as the other two diets, it should have performed even more favorably [31]. To this point, it is noteworthy that POL, with its superior metabolic effect, had the lowest total dietary 18:2 content and only 1% of its TG molecules having sn2-18:2 with most 18:2 at sn1,3. These collective data imply that a lack of 18:2 at sn2 is not as problematic as the abnormal insertion of SFA (or TFA) in that location.

These data also support the previous conclusion [30] that TFA are more detrimental than either of the two main SFA (16:0 and 18:0) that they were designed to replace in food products, at least gram for gram when considering lipoprotein metabolism. It is also clear that SFA as 16:0 in the TG-MS of natural POL was an improvement (even with less 18:2 present) compared to an approximately equal mass of 18:0 randomly inserted into SBO in the form of the IE fat, leading to a significant amount of 18:0 at sn2. This indicates that 18:0 should not be considered a neutral SFA, at least when randomized into sn2 or if it becomes the major dietary SFA. By the same token, others have shown that 16:0 randomized into the sn2 position raises LDL cholesterol in men when compared to natural palm oil [10], a fat in which sn2 is largely *cis*18:1, similar to the situation with POL here. Thus, as these several studies demonstrate, manipulating dietary TG-MS can negatively influence plasma lipoproteins, even though the exact degree of TG modification required and the mechanism are unclear.

Modifying sn2 fatty acids, particularly by introducing a saturated fatty acid at this site, would seem a likely candidate for distorting lipoprotein metabolism. Caprenin, an artificial fat with randomized behenic acid (22:0), exerts a negative impact on human LDL/HDL metabolism similar to that seen with TFA [32], and both 22:0 and TFA elicited effects similar to randomized 18:0 observed in this study and in two previous studies where IE fat was fed [8,9]. Long chain (18-22C) saturated fatty acids are relatively uncommon in natural fats, with 18:0 representing the most prevalent at intakes <2–4% daily energy. Furthermore, 18:0 in natural fats is usually esterified at sn1 and sn3 on glycerol, as in beef tallow and cocoabutter [33]. Artificial insertion of 18:0 at sn2 during random interesterification, along with the shear mass of 18:0 consumed (12 %en), may have been problematic in the current study and previously [8,9]. The mass of 18:0 consumed may be critical because a large intake (7 %en), from sheanut butter was found to depress HDL similar to Caprenin [34]. Sheanut butter is reported to have 18% of sn2 as SFA (mostly 18:0), or 3× the amount of sn2-SFA present in cocoabutter [33], a fat that does not appear to affect lipoprotein metabolism adversely [4,12]. Although consumption of 12 %en as 18:0 would not be feasible from natural fats, it is possible to envision an exaggerated intake via structurally modified fats resulting from the growing impetus to eliminate TFA from the diet. The incorporation of 18:0 has seemed especially appealing since 18:0 from natural fats often has been considered neutral in its impact on cholesterol metabolism [2-4,35].

### Glucose perturbations

Among studies on the metabolic effects of randomized fat involving 18:0, metabolism of glucose and insulin has not been addressed. Considering global trends in obesity, insulin resistance, and diabetes, which are often associated with the metabolic syndrome, our observation that altered dietary TG-MS may adversely influence glucose metabolism warrants attention. From epidemiological data on the association between TFA intake and diabetes [36] one might have anticipated the TFA-induced rise in glucose observed after 4 wk. Even though previous experiments involving TFA in humans found no effect on glucose metabolism other than postprandial insulin hypersecretion [37,38], those studies reported essentially no change in the fasting lipoprotein profile either, which is somewhat atypical. In our study IE fat had less effect than the TFA-rich PHSO on lipoproteins, but proved more deleterious for glucose metabolism, suggesting that modified TG-MS, from either trans-rich or IE fats, was a factor.

After 4 wk on each diet, fasting insulin was inversely related to glucose, ie. insulin was moderately lower after PHSO (-10%, ns) and substantially lower after IE (-20%, p < 0.001) compared to POL, while glucose was significantly elevated by both modified fats (about 5% and 20%, respectively). The elevated 4 wk fasting glucose following IE was foreshadowed by the 40% greater glucose IAUC observed during the 8 h postprandial challenge with the IE test meal. The patterns of lower plasma insulin (IE, PHSO) and C-peptide (IE) postprandially following the test meal challenge with the two modified fats relative to POL, suggests that reduced insulin secretion, rather than insulin resistance, accounted for the higher glucose values observed with modified fats. The 20% rise in fasting glucose with IE was clinically important, as well, since it rose to a range that could be considered prediabetic after only 4 wk [39]. These results appear to be in contrast to the elevated serum insulin and insulin resistance typical of obesity and type 2 diabetes associated with the metabolic syndrome [40,41].

Our findings suggest that altered dietary fat composition or TG-MS influenced insulin secretion to impact glucose metabolism negatively. Others have reported initially lower blood glucose coupled with sharply higher initial insulin secretion 20–60 min postprandially in subjects fed a single meal of IE palm oil compared to natural palm oil [11]. Postprandial insulin hypersecretion also occurred in diabetic subjects fed extreme intakes of TFA or SFA compared to cisMUFA [38]. Thus, a connection seems to exist between dietary fat composition and insulin secretion. The link may include the n-3 PUFA content of fats. For example, it is noteworthy that dietary 18:3n-3 and long chain n-3 PUFA have been linked to production of intestinal GLP-1 [42], which enhances insulin secretion. It may prove ironic that partial hydrogenation of vegetable oils, or interesterification with 18:0, are implemented in part to remove 18:3n-3 and improve product shelf life, even though fats modified in this manner may inadvertently suppress insulin secretion after their prolonged consumption. Interesterification resulting in high intake of 18:0, including sn2-18:0, appeared to accentuate the negative effect observed with the TFA diet. In reference to insulin metabolism, both 18:0 and TFA fed at high levels (8% en) also induced inflammatory markers [43] that are associated with diabetes [44].

It is not apparent whether the observed alteration in glucose and lipoprotein metabolism represents a fatty acid effect per se or modification of TG-MS. For example, earlier work in gerbils traced saturated fat intake and inositol deficiency to a lack of PUFA needed structurally for phosphatidyl inostitol (PI) synthesis required for fat secretion by intestinal mucosal cells [45]. Since PI is also involved in beta-cell secretion of insulin [46], PI structure or availability may be a consideration in the mechanistic aspect of our current clinical observations. Extreme SFA consumption (usually to the exclusion of dietary PUFA) is typically linked to insulin resistance [47], while other studies have reported that dietary SFA enhances insulin secretion more than dietary PUFA [48], a possibility suggested by our findings where POL diet had the lowest P/S ratio. Clarification is needed, but careful scrutiny of the dietary PUFA load as it affects dietary TG-MS and phospholipid synthesis related to insulin dynamics is warranted [6].

In past investigations, glucose metabolism has not received the intense scrutiny afforded lipoproteins in most studies involving dietary fat. The present results linking TFA and 18:0- rich IE fats with abnormal metabolism of glucose suggest that it would be prudent to determine the biologically tolerable mass of 18:0 that can be incorporated in diets as IE fats.

## Conclusion

A natural saturated fat, palm olein, was compared to two forms of modified replacement fats, one containing *trans *fatty acids, the other interesterified with 18:0. Both modified fats adversely altered metabolism of plasma lipoproteins and blood glucose in humans. Further investigation is warranted before interesterification is disseminated as the process of choice for replacing partial hydrogenation as a primary means for hardening vegetable oils for use in foods.

## Abbreviations

BMI, body mass index; FA, fatty acid; IE, interesterified fat; IAUC, integrated area under the curve; MUFA, monounsaturated fatty acids; PHSO, partially hydrogenated soybean oil; PI, phosphatidyl inositol; POL, palm olein; PUFA, polyunsaturated fatty acids; SFA, saturated fatty acids; TC, total plasma cholesterol; TFA, *trans *fatty acids; TG, triacylglycerol; TG-MS, triacylglycerol-molecular species;

## Competing interests

Financial support for this study was provided by the Malaysian Palm Oil Board where Dr Sundram was employed and Dr Karupaiah was a graduate student. Dr Hayes is a member of the Malaysian Palm Oil Advisory Council.
